# Transbronchial lymph node forceps biopsy as a novel tool in diagnosis of mediastinal lymphadenopathy: a pilot study

**DOI:** 10.1186/s13019-024-02560-x

**Published:** 2024-02-07

**Authors:** Ahmed Al-Halfawy, Sabah Hussein, Wafaa Ashur, Ali El-Hendawi, Sara Hussein

**Affiliations:** 1 Faculty of Medicine, Cairo University, Giza, Egypt; 2 Giza Chest Hospital, Giza Governorate, Egypt

**Keywords:** C-TBNA, LN-TBFB, Mediastinal Lymphadenopathy

## Abstract

**Background:**

Differential diagnosis of mediastinal lymphadenopathy is an issue of debate. Lymph nodes may be enlarged due to a variety of inflammatory, infectious, or malignant reasons. Therefore, obtaining samples from the affected nodes is crucial for the diagnosis. Usually, these patients are subjected to TBNA (EBUS or conventional) or mediastinoscopy if TBNA is not conclusive. This study evaluated the safety and feasibility of this new technique of transbronchial forceps biopsy for the diagnosis of mediastinal lymphadenopathy.

**Methods:**

The study included 18 patients with confirmed mediastinal lymphadenopathy who were admitted in Chest Department, Cairo University in the period from December 2019 to December 2020. All patients were subjected to flexible bronchoscopy with conventional transbronchial needle aspiration (C-TBNA) and transbronchial forceps biopsy (LN-TBFB) from the enlarged mediastinal lymph node in the same procedure.

**Results:**

we found the technique of LN-TBFB safe with no serious complications. We were able to reach a diagnosis in 7/7 (100%) cases of sarcoidosis, 6/7 (85.7%) cases of malignant lymph nodes. We had three cases where the histopathology showed hyperactive follicular hyperplasia, and a single case of tuberculous lymphadenitis. C-TBNA was diagnostic in 71.4% of sarcoidosis cases, 42.9% of malignant cases, but failed to diagnose the one patient with tuberculous lymphadenitis.

**Conclusion:**

Lymph node transbronchial forceps biopsy (LN-TBFB) was found to be safe and effective in the diagnosis of mediastinal lymphadenopathy. We strongly advocate the use of this minimally invasive technique for diagnosing pathologically enlarged mediastinal lymph nodes, as a last step before mediastinoscopy.

## Introduction

The differential diagnosis of mediastinal lymphadenopathy is an issue of debate. Lymph nodes may be enlarged due to variety of inflammatory, infectious, or malignant reasons. The diagnosis in cases of mediastinal and /or hilar lymphadenopathy with no lung parenchymal involvement is often difficult and requires obtaining specimen from the enlarged lymph nodes [1]. 

Conventional transbronchial needle aspiration (C-TBNA) provides an opportunity to diagnose mediastinal lesions and to stage bronchogenic carcinoma in a minimally invasive fashion [2]. The sensitivity of C-TBNA ranges from 69 to 90% but the sensitivity ranges from 37–89% [3]. Endobronchial ultrasound-guided transbronchial needle aspiration (EBUS-TBNA) is considered the investigation of choice for sampling mediastinal nodes. A major drawback of EBUS-TBNA is its lower diagnostic yield for lymphoma and benign diseases [4] as well as its high cost especially in low income countries.

Lymph node transbronchial forceps biopsy (LN-TBFB) is a novel bronchoscopic sampling technique to improve the capability of obtaining tissue for histological examination from mediastinal lesions. LN-TBFB is safe and provides adequate histological specimens of enlarged mediastinal lymph nodes. LN-TBFB increases the diagnostic yield in benign conditions and may add value in molecular analysis of non-small cell lung cancer [5]. 

This pilot study was carried out to investigate the safety and feasibility of lymph node transbronchial forceps biopsy (LN-TBFB) as a new tool for diagnosis of mediastinal lymphadenopathy in the different etiologies.

## Materials & methods

This pilot study included 18 patients with confirmed mediastinal lymphadenopathy as shown by CT chest with IV contrast who were admitted in Chest Department during the period from December 2019 to December 2020. The study was performed in collaboration with the Pathology Department. The Institutional Research Ethics Committee, has approved the study in December 2019 (code: MD-202-2019).

All patients were informed with the full details of the procedure and a written informed consent was obtained. All patients older than 18 years old of both sexes who had mediastinal lymphadenopathy on contrast enhanced CT chest were included in the study. Exclusion criteria included patient refusal to give informed consent, patients with coagulation defects (prothrombin concentration should be > 60% and the platelets count should be > 60,000/mm^3^), major organ failure (heart failure, liver cell failure, renal failure), unstable Arrhythmia, recent myocardial infarction (< 6 weeks duration) & patients with respiratory failure.

### Methodology in details

All patients were subjected to complete history taking and detailed clinical examination, routine labs (CBC, liver & kidney function, blood sugar, bleeding profile), tuberculin skin test, computed tomography chest with IV contrast. Then, bronchoscopy was performed to obtain conventional transbronchial needle aspirate (C-TBNA) and lymph node transbronchial forceps biopsy (LN-TBFB).

**Fig. 1:**
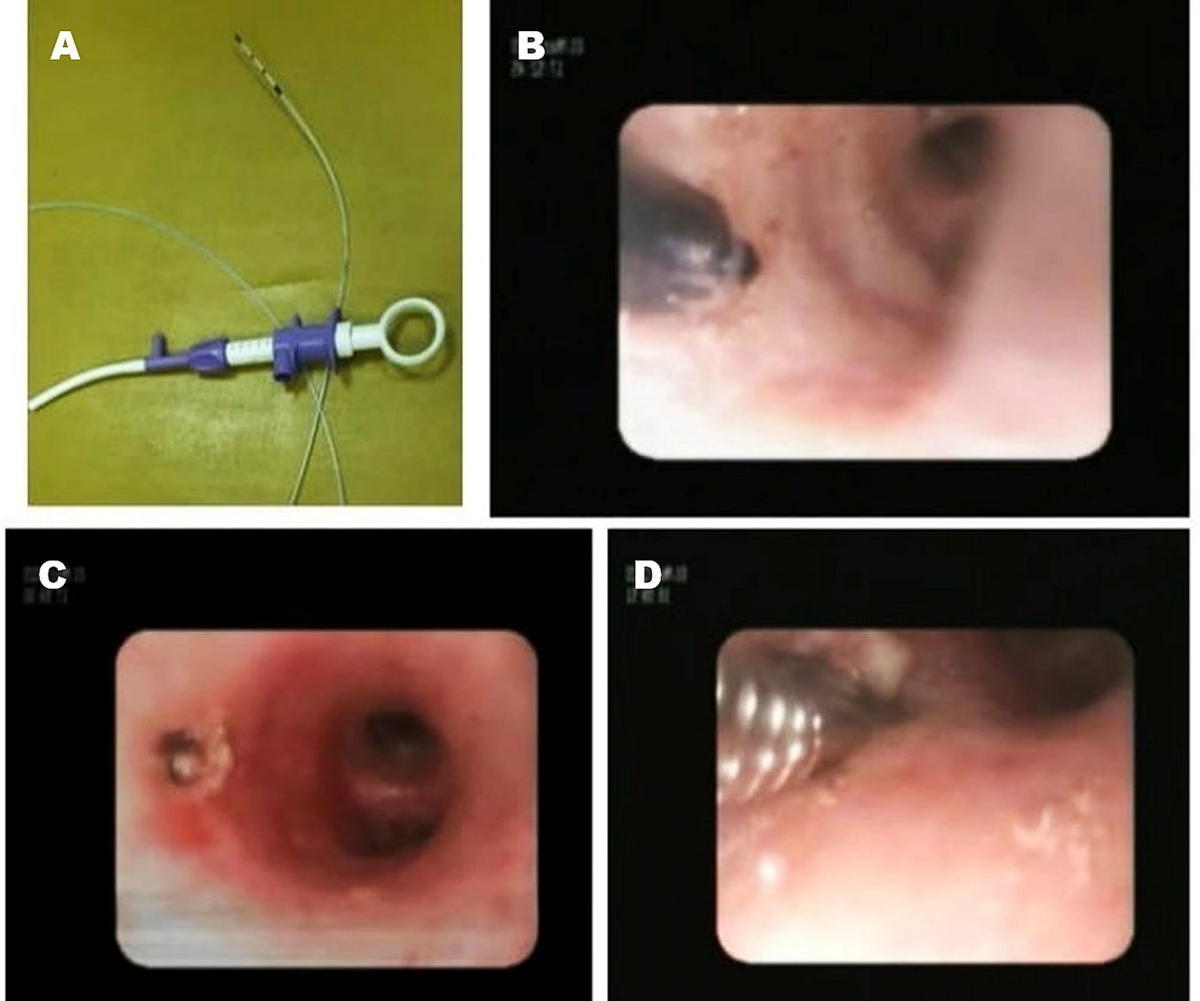
(**A**) Endoflex needle knife. (**B**) Needle knife penetration through the bronchial wall. (**C**) the hole created in the bronchial wall. (**D**) the 1.8 mm forceps passed through the hole in the bronchial wall. Transbronchial forceps was advanced through incision and biopsy was obtained

- **patient Preparation & pre-medications**:
  Patients taking oral anticoagulation were asked to stop the medications 5 days prior to the procedure. We administered pre-procedure Atropine to minimize secretions [6]. Conscious sedation with midazolam infusion was maintained throughout the procedure. Lidocaine solution 2% was used as local anesthetic.
- **conventional transbronchial needle aspirate (C-TBNA)**:
  After completing inspection of the bronchial tree, c-TBNA was performed at the site of the largest lymph node station. We used the jabbing technique. Material obtained was smeared on a slide and fixed with 95% alcohol, then stained with Papanicolaou stain and hematoxylin and eosin (H& E) stain [7].
- **lymph node transbronchial forceps biopsy (LN-TBFB)**:
  In the same site of blind C-TBNA, a 2 mm incision was done using endoflex needle knife (Fig. 1) connected to electrocautery device (ERBE VIO 300s-Electrosurgical unit) at 20–40 W of energy. The incision was in the inter-cartilaginous space, penetrating the whole airway wall. Then the 1.8 mm forceps was advanced through the incision and transbronchial forceps biopsies were obtained. As the technique is still investigative, and no EBUS was used, we confined our LN-TBFB to LN stations 4 and 7 only to minimize risk of bleeding. (Fig. 1). The biopsy specimens were preserved in formalin containing cups and sent for histopathological examination.
- **potential risks**:
  We anticipated possible complications of this new technique such as bleeding, pneumothorax, or pneumomediastinum.
- **statistical methods**:
  Data analysis packages SPSS version 21 was used for data analysis. Qualitative data were presented by number and percentage, quantitative data were presented by mean, standard deviation, median and interquartile range. Parametric and non-parametric tests of significance were done. Chi square test with Fischer exact test was used for non-parametric data. Sensitivity was calculated. Level of significance was set at p equal to or below 0.05.

## Results

This is a pilot study that included 18 patients with mediastinal lymphadenopathy. Their mean age was 45.3 ± 14.9 years. Their demographic data is shown in Table (1). On CT-scan of the chest, the enlarged lymph node stations, characters and associated radiological lesions in our patients are shown in Table (2). Stage 4R was present in all of our patients, while the subcarinal group of lymph nodes were present in 17/18 patients (94.4%). In our case series, there were 7 patients whose final diagnosis was sarcoidosis, 7 patients with malignancy (3 small cell lung cancer, 2 non-small cell cancer, and 2 cases of lymphoma). We encountered one case of tuberculous lymphadenitis, and three cases of reactive hyperplasia. The final histological diagnoses obtained from the c-TBNA and LN-TBFB are shown in Table (3).

**Table 1:** Patients demographics

Mean age	45.3 ± 14.9 years		
Gender	Male Female	9 9	50% 50%
Smoking history	Smoker Non-smoker	9 9	50% 50%
Comorbidities:	Hypertension IHD Diabetes Mellitus Peptic ulcer	4 3 3 1	22.2% 16.7% 16.7% 5.6%

**Table 2:** The affected mediastinal lymph node stations, characters & associated radiological lesions

Lymph node station	Number	%
2 R	3	16.7%
2 L	2	11.1%
4 R	18	100%
4 L	11	61.1%
5	3	16.7%
6	8	44.4%
7	15	93.3%
10 R	5	27.7%
10 L	2	11.1%
Bilateral hilar	11	61.1%
Peripheral LN	2	11.1%
Lymph node character	Number	%
Discrete	15	83.3
Amalgamated	3	16.7
Associated radiological lesions	Number	%
Lung nodules	11	61.1
Ground glass opacification	7	38.9
Reticulations	6	33.3
Lung mass	1	5.6
Pleural effusion	1	5.6
Pericardial effusion	1	5.6

**Table 3:** Diagnostic yield of c-TBNA and LN-TBFB

	c-TBNA		LN-TBFB	
Sarcoidosis	5/7	27.7%	7/7	38.8%
Malignancy	3/7	16.6%	6/7	38.8%
Tuberculosis	1/1	16.6%	1/1	5.5%
non-specific	3/3	16.6%	3/3	16.6%
Total	12/18	66.6%	17/18	94.4%

**Fig. 2:**
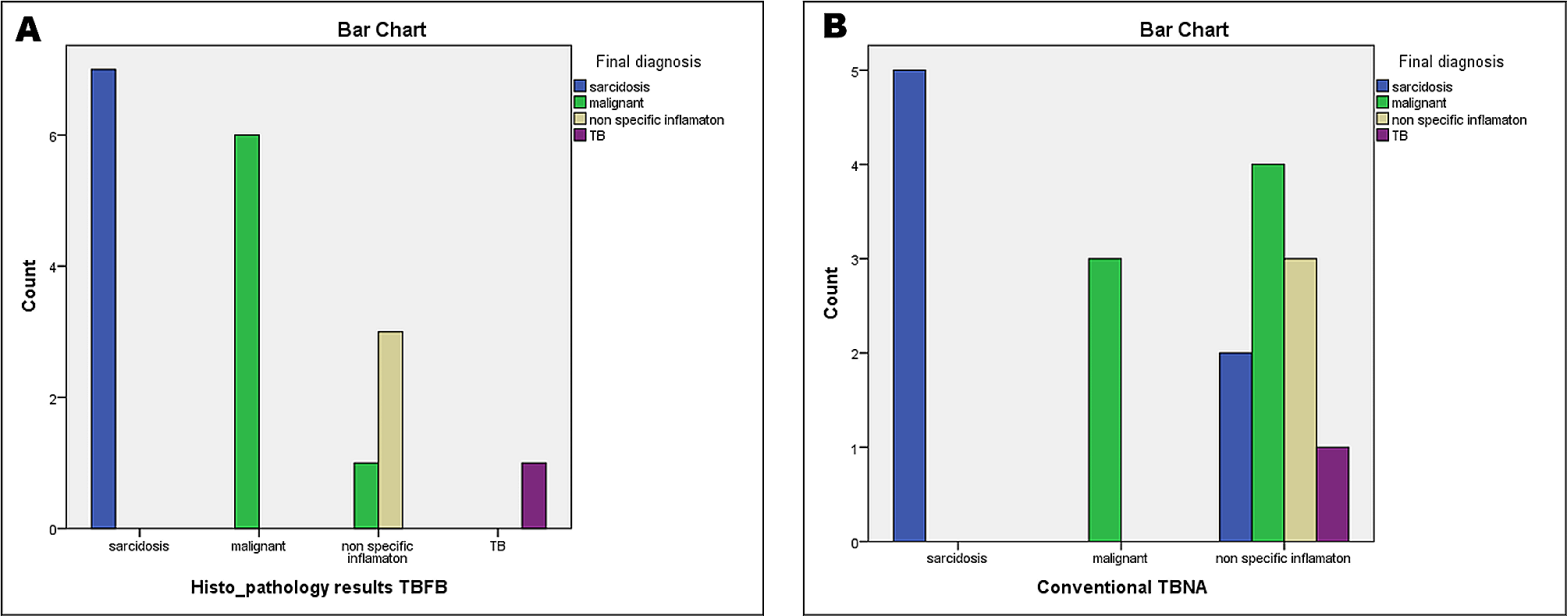
Bar Chart showing LN-TBFB histopathology results to final diagnosis crosstabulation among the studied patients with mediastinal lymphadenopathy (**A**). Conventional TBNA cytopathology results to final diagnosis crosstabulation among the studied patients with mediastinal lymphadenopathy (**B**)

The diagnostic yield of c-TBNA in our series was 66.6% in different etiologies, while the diagnostic yield for the LN-TBFB was 94.4%. LN-TBFB was able to reach the diagnosis of sarcoidosis in 7/7 patients (100%). The technique was also able to diagnose 6 cases of malignancy out of 7 (85.7%). One patient was proved to be a case of lymphoma after the patient underwent mediastinoscopy, after an inconclusive LN-TBFB. In 6 instances, LN-TBFB histopathology was diagnostic when C-TBNA cytopathology was non-diagnostic (Fig. 2). The procedure was safe, we encountered no pneumothoraces or pneumomediastinum. The procedure resulted in minor bleeding, similar to that observed during a normal bronchial biopsy. In one case, the bleeding was more profuse and < 50 ml of blood was aspirated. Simple tamponading of the electrocautery incision by the tip of the scope, along with cold saline wash controlled the bleeding instantaneously. Patients remained under observation overnight and then were discharged after clinical and radiological examinations were satisfactory.

## Discussion

Mediastinal and hilar lymphadenopathy is a common condition, and according to the American College of Chest Physicians (ACCP) guidelines, endobronchial ultrasound (EBUS) transbronchial needle aspiration (TBNA) is considered standard of care for tissue acquisition in such patients. Its yield varies depending on the clinical scenario [8]. After nondiagnostic EBUS-TBNA, mediastinoscopy is often recommended, although it incurs more length of stay, complications, and higher costs [9]. Our new technique may be used in those cases of mediastinal lymphadenopathy after a non-conclusive TBNA (conventional or EBUS-guided) or in the centers where EBUS is not readily available. It has proved safe, simple, and requires no additional equipment than those in the regular bronchoscopy suite.

The diagnostic efficiency of the various biopsy methods remains controversial. The reported diagnostic rate of C-TBNA in patients with benign mediastinal lymph node enlargement varies between 21.4 and 76% [10], while that of EBUS-TBNA varies between 74.5 and 96% [11, 12]. Although EBUS improves the diagnostic rate of TBNA, puncture specimens are usually a mixture of cell masses and blood clots that are not always morphologically representative. A few studies have introduced the technique of lymph node transbronchial forceps biopsy (LN-TBFB) as a potential option to obtain a large volume of biopsy specimen, which could aid in the diagnosis of mediastinal lymphadenopathy. One of the earliest descriptions of similar techniques was published by Oki et al. in 2004, where they used a 19-gauge TBNA needle to puncture the bronchial wall and then inserted a mini forceps to obtain biopsies from the mediastinal lymph nodes [13–15].

In our case series, we tested the efficacy and safety of lymph node transbronchial forceps biopsy (LN-TBFB) using the electrocautery device and a regular biopsy forceps, as a new tool for diagnosis of the mediastinal lymph nodes enlargement.

In our study, the mean age of patients was 45.3 ± 14.9 years, 50% of them (9/18) were male smokers. The groups that we attempted were the R4 station which was enlarged in all of our patients, or station 7 which was enlarged in 93.3% of cases.

There were attempts at obtaining forceps samples from mediastinal LNs by other investigators. ***Rüber et al.*** [16] compared the safety, and yield of EBUS-TBFB using the 1.5 mm mini-forceps versus EBUS-TBNA alone. The overall DY was 61.9% and 85.7% for TBNA alone and EBUS-TBNA combined with EBUS-TBFB. Also ***kim et al.*** [17], investigated the same technique of EBUS-TBFB using electrocautery with similar results.

**Fig. 3:**
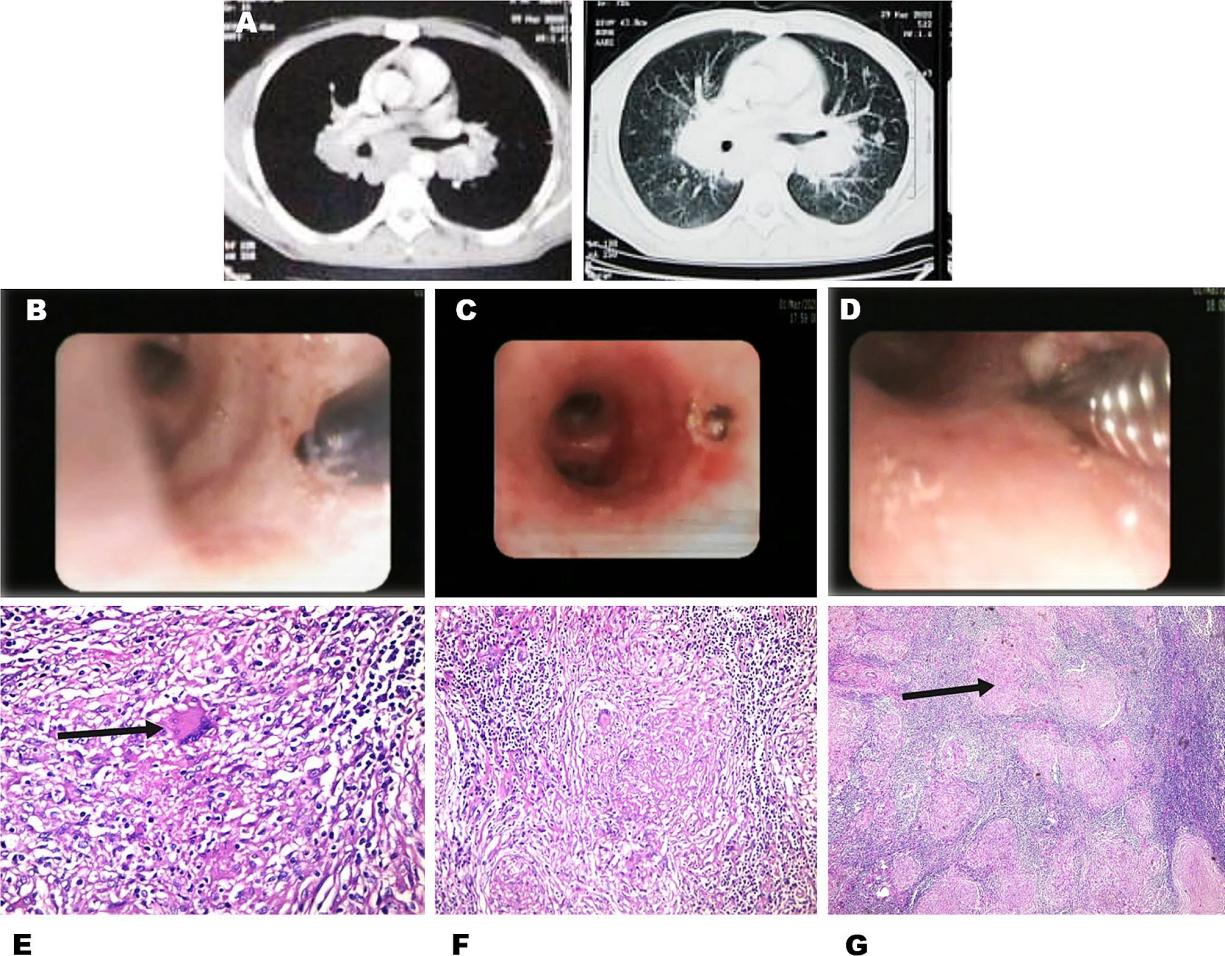
26 years old male, presenting with dry cough & dyspnea. CT chest with IV contrast (**A**) showing subcarinal and bilateral hilar discrete L.N with parenchymal nodules. The needle knife (**B**) was used to create the hole inside the bronchial wall (**C**). The forceps was advanced through the incision (**D**) and LN-TBFB was obtained from the enlarged subcarinal L.N. LN-TBFB image (high power) (**E**) showing multinucleated giant cells (black arrow) support the diagnosis of sarcoidosis. LN

**Fig. 4:**
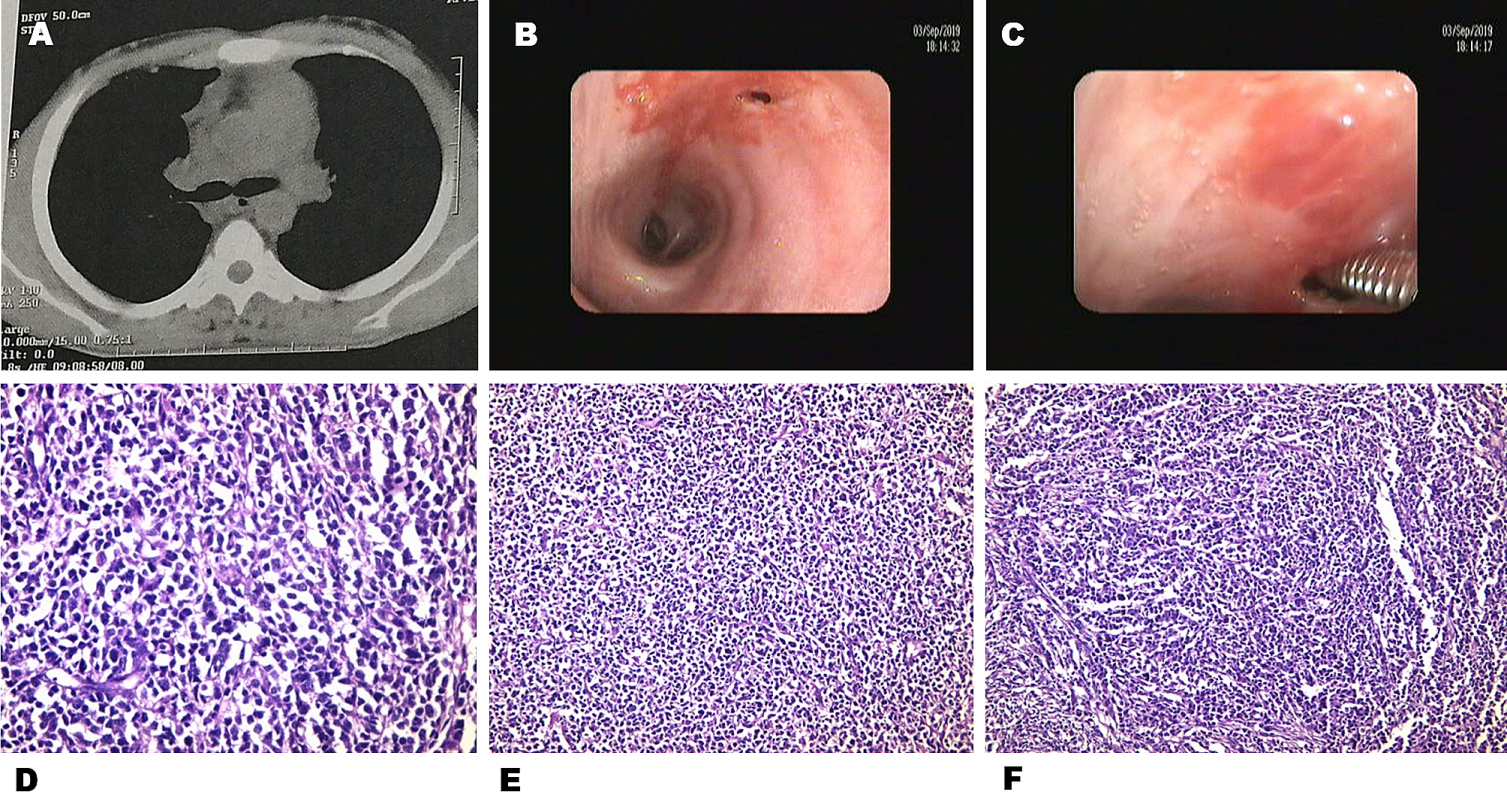
21 years old male, presenting with oedema of the face & upper limb with severe dyspnea. CT chest mediastinal window (**A**) showing subcarinal & bilateral hilar, discrete mediastinal lymph nodes. The forceps was advanced through the incision made in the bronchial wall (**B, C**) and LN-TBFBs was obtained from subcarinal L.N. LN TBFB image (high power) (**D**), (intermediate power) (**E**) & LN-TBFB image (low power) (**F**) showing atypical round cells (a case of large cell lymphoma)

In the current study, biopsy results of LN-TBFB were 100% diagnostic in cases of sarcoidosis (Fig. 3) and tuberculosis. Malignancy was diagnosed in 85.7% of cases (6/7) including 3 cases who were diagnosed as small cell lung cancer, 2 cases who were diagnosed as non-small cell cancer and 1 case who was diagnosed as lymphoma (Fig. 4). However, malignancy was misdiagnosed in one case (14.3%) which proved later to be a case of lymphoma by mediastinoscopy (*P* = 0.000). The sensitivity of LN-TBFB was 94.4%. C-TBNA cytopathology results were diagnostic only in 71.4% of sarcoidosis (5/7), 42.9% of malignancy (3/7) and was inconclusive in the one case of TB lymphadenitis (*P* = 0.000). The sensitivity of C-TBNA was 61.1%. In 6 instances, LN TBFB histopathology was diagnostic when C-TBNA cytopathology was non-diagnostic (Fig. 2). There were no reported complications using the LN-TBFB method in the present study. Our results were in accordance with ***Rüber et al.*** [16] who stated that the overall diagnostic yield was 61.9% and 85.7% for TBNA alone and EBUS-TBNA combined with EBUS-TBFB, respectively (*p* < 0.001). The combined approach in their study was associated with a significantly higher diagnostic yield for lung cancer diagnosis (97.1% vs. 76.5%, *p* = 0.016) and sarcoidosis (85.2% vs. 44.4%, *p* = 0.001) and no major adverse events were observed.

Similarly, ***Darwiche et al.*** [14] were able to obtain adequate samples to test for Epidermal growth factor receptor (EGFR) mutation, which was not possible with c-TBNA slides. This study has some limitations such as a small study cohort in a single center.

In conclusion, we have found that lymph node transbronchial forceps biopsy (LN-TBFB) is safe and effective in diagnosing mediastinal lymphadenopathy. We understand that the number of cases in our series is a major limitation. However, considering the higher diagnostic yield, the low rate of complications, and the cost effectiveness of the technique, it may be suitable for those centers where EBUS is not available, or when tissue samples rather than cytology specimens are required for the diagnosis of different etiologies.

## Data Availability

Data supporting reported results are available and can be found.
